# Clinical and audiological profile of patients with sudden sensorineural hearing loss after exposure to recreational noise

**DOI:** 10.1016/j.bjorl.2025.101617

**Published:** 2025-04-10

**Authors:** Lara Freire Bezerril Soares, Breno Lima de Almeida, Igor Ataíde Silva Teixeira, Maria Luisa Frechiani Lara Maciel, Jorge Vinícius Leocádio Monteiro, Luanna Miranda Martins, Marina Cançado Passarelli Scott, Norma de Oliveira Penido

**Affiliations:** a Universidade Federal de São Paulo (UNIFESP), Departamento de Otorrinolaringologia e Cirurgia de Cabeça e Pescoço, São Paulo, SP, Brazil; b Universidade Nove de Julho (UNINOVE), São Paulo, SP, Brazil

**Keywords:** Sensorineural hearing loss, Sudden hearing loss, Noise-induced hearing loss, Hearing loss

## Abstract

According to the World Health Organization, significant hearing loss in young people exposed to recreational acoustic trauma has become a public health issue.

**Objective:**

To describe the clinical and audiological profile of individuals with Sudden Sensorineural Hearing Loss (SSNHL) exposed to recreational noise. Methods. A prospective cohort study was conducted involving outpatients with SSNHL and a history of exposure to acute recreational noise. Follow-up occurred weekly in the first month, then monthly for up to six months of follow-up. All were treated with oral corticosteroids with prednisone 1 mg/kg/day, followed by a gradual dose reduction of 20 mg every 7-days until complete withdrawal of the medication.

**Results:**

Twelve individuals were included, 8 (66.7%) males, with a mean age of 24.16-years. 58.3% of the individuals had binaural involvement. Only one patient had permanent profound hearing loss, with no sign of recovery after treatment. All had associated tinnitus. Half of the participants reported having used recreational drugs, and the type of exposure varied from New Year's Eve parties, funk and electronic music, concerts and games in stadiums. Regarding the duration of exposure and the distance from the sound source, most reported a time of 3–5 h, with a short distance from the source.

**Discussion:**

Recreational noise exposures have the potential to increase the risk of hearing loss in individuals outside of the workplace. Data such as sex, age group and the most prevalent type of noise exposure in these cases reported in this study corroborate the findings in the literature. Regarding the persistence of hearing loss after treatment, the outcome was varied, and the studies are still controversial.

**Conclusion:**

There is an association between exposure to recreational noise and SSNHL, and the epidemiological profile of patients affected by this condition is marked by adolescents and young male adults with unilateral and binaural involvement, severe degree of hearing loss at onset, and commonly associated with the use of recreational drugs, risking permanent hearing damage.

**Level of evidence:**

3 (OCEBM Levels of Evidence Working Group*. "The Oxford 2011 Levels of Evidence").

## Highlights

• Recreational acoustic trauma is a growing public health concern in young people.
• Profiles of young adults with sudden hearing loss after noise exposure are described.
• Noise exposure included music events and parties for 3–5 h near sound sources.
• Recreational drug use is common and harms hearing health, alongside noise exposure.
• Significant auditory sequelae may occur due to variable rates of hearing recovery.

## Introduction

Noise damages the auditory system through two distinct mechanisms. The first is when an individual is exposed to an intense noise peak, greater than 140 dB, which can cause immediate and often permanent hearing loss. This is called acoustic trauma and occurs when the structures of the inner ear are stretched beyond their limits. In contrast to this type of acute mechanical injury, chronic exposure to noise between 90 and 140 dB results in permanent metabolic cochlear damage, called Noise-Induced Hearing Loss (NIHL).^1^, ^2^

Acoustic trauma is a well-established condition which results from exposure to intense noise, whether sudden or continuous, and manifests as tinnitus, otalgia and hearing loss, depending on its severity.^3^ NIHL, in turn, is defined as a decline in auditory function secondary to chronic exposure to high-intensity sounds, whether occupational or recreational.^4^, ^5^ Hearing loss is usually sensorineural and bilateral.^6^

Sudden Sensorineural Hearing Loss (SSNHL) is also a well-defined symptom, characterized as sensorineural hearing loss of at least 30 dB in 3 consecutive frequencies for at least 72 h. Several causes have been proposed, including tumors, immune-mediated diseases, viral infections and NIHL.^7^

However, little is known about SSNHL induced by recreational noise. Recreational activities are a major source of inducing hearing loss,^8^ not only in the long term, but also acutely. There are reports of clinical studies since 1800 documenting hearing loss after exposure to gunshots, for example.^9^

An increase in hearing impairment in children and adolescents related to exposure to leisure noise has been described.^10^ The World Health Organization estimates that 1.1 billion young people worldwide may be at risk of hearing loss due to indiscriminate exposure to high-intensity noise, especially considering the habits of listening to music in bars, nightclubs, parties and the use of insert earphones.^11^

The consumption of alcohol, cigarettes and drugs are another concern in this age group. Smoking can impact the auditory system through direct ototoxic effects of nicotine or other ototoxic substances found in cigarette smoke, or through vascular effects, such as increased blood viscosity and reduced available oxygen, leading to cochlear hypoxia.^12^, ^13^, ^14^ The association between these kinds of consumptions abovementioned and exposure to high-intensity noise may therefore have a potential effect on damage to the inner ear.

The present study aims to describe the clinical and audiological profile of individuals with SSNHL exposed to recreational noise.

## Methods

A prospective cohort study was carried out involving patients from the SSNHL outpatient clinic of Universidade Federal de São Paulo/Escola Paulista de Medicina in the period between March 2020 and February 2024. Patients of both sexes with a diagnosis of SSNHL and a history of exposure to recreational noise, confirmed by pure tone testing, speech audiometry and immittance audiometry, according to the criteria established by the American Academy of Otolaryngology – Head and Neck Surgery (AAO-HNS), were included.

### Ethical considerations

The study was approved by the Ethics Committee of Universidade Federal de São Paulo/Escola Paulista de Medicina under the statement 1466/2020. Written informed consent forms were applied to study participants. For individuals under 18 years of age, the informed consent form was applied and completed by their respective guardians.

### Inclusion criteria

Patients diagnosed with SSNHL, confirmed by audiological examination, undergoing follow-up at the otology clinic for clinical and audiological investigation and who had a history of exposure to high-pitched recreational noise. Absence of cognitive alterations that prevent collaboration with clinical history and audiometric examinations.

### Exclusion criteria

Patients with other audiometric configurations not consistent with a diagnosis of SSNHL (conductive hearing loss, mixed hearing loss, rapidly progressive or progressive), or the patient's refusal to participate in the study.

### Clinical evaluation

Patients underwent structured anamnesis, with assessment of demographic data and otorhinolaryngological physical examination, carried out in pre-established routine consultations after initial care, with weekly frequency in the first month, then monthly up to six months of follow-up.

### Audiometric evaluation

The diagnosis was confirmed based on the AAO-HNS criteria, considering the definition of SSNHL, with a threshold reduction of 30 dB or more, in at least three contiguous audiometric frequencies, occurring over a 72 -h time window. An annually calibrated Interacoustics AZ7 audiometer was used to assess air conduction hearing thresholds in frequencies from 250 Hz to 8000 Hz, bone conduction from 500 Hz to 4000 Hz, and to perform vocal audiometry, Speech Recognition Threshold (SRT) and Speech Recognition Percentage Index (SRPI).

The masking used was Speech Noise and Narrow Band. Immittance analysis was also performed. The audiometric examination was performed at the time of suspected diagnosis, followed by new examinations at pre-established intervals of 7-, 30- and 120-days. The Pure Tone Average (PTA) was calculated using air conduction thresholds of 500-, 1000-, 2000- and 4000-Hz. The PTA value for bilateral cases was calculated after performing the arithmetic mean between the two affected ears. Hearing loss was measured according to the WHO grading (WHO 2020): no impairment <20 dB, mild between 20 and 34 dB, moderate between 35 and 49 dB, moderately severe, 50–64 dB, severe between 65 and 79 dB, profound impairment between 80 and 94 dB, complete hearing loss >94 dB. The audiogram configuration was based on the Silman and Silverman (1997) classification adapted from Carhart (1945) and Lloyd and Kaplan (1978). The criteria for symmetry or asymmetry were based on the American Speech Language Hearing Association (ASHA 2011) recommendations.

Hearing recovery criteria were based on the AAO-HNS guidelines for cases of unilateral hearing loss with normal contralateral hearing. For bilateral cases or contralateral hearing with previous loss (unrelated to the current event), we considered complete recovery when the final PTA value was above 20 dB, a level considered normal hearing according to the WHO criteria (2020), or when returning to previous hearing, regardless of the degree, if an audiometric examination prior to the loss was available. Partial recovery was considered in subcategories, classified as partial, significant partial, and non-significant partial (understood in the AAO-HNS classification), depending on the degree of hearing loss at the time of sudden deafness, the amplitude of recovery, and the final auditory impact. Cases with a variation of less than 10 dB were considered as having no recovery. In individuals with hearing worsening, we considered any drop of at least 10 dB in the PTA and a change to worsening in the criterion for assessing the functional auditory degree. Regarding the hearing recovery criteria, we considered patients with hearing improvement, to some degree, classified as complete, partial and significant partial recovery. Without hearing improvement, patients were classified as having no significant recovery or no recovery.

### Treatment

All patients included in the study were treated with oral corticosteroids (prednisolone 1 mg/kg/day in a single daily dose, maximum dose 60 mg/day, for 14 days, followed by gradual dose reduction of 20 mg every 7 days, until complete withdrawal of the medication). If the follow-up audiogram in the first week had shown complete hearing recovery, dose reduction began on day 7, not on day 14. In this study, rescue therapy with intratympanic corticosteroids was not used.

## Results

A total of 80 individuals diagnosed with sudden sensorineural hearing loss were evaluated between March 2020 and February 2024. Of these, 12 (15%) had exposure to recreational noise. All patients had a diagnosis of sudden deafness confirmed by audiometry.

Of the 12, 8 (66.7%) were male and 4 (33.3%) were female, with a mean age of 24.16 years, all without associated comorbidities. Regarding hearing loss laterality, 58.3% of the patients had both ears affected (bilateral loss), while the other 41.6% had unilateral loss. One patient had permanent profound hearing loss, with no sign of hearing recovery after treatment, and was referred for cochlear implant surgery; the others had moderate, moderately severe and severe hearing loss. 100% of the patients presented associated tinnitus (Table 1).

**Table 1:** Clinical and audiological profile of patients with sudden sensorineural hearing loss after exposure to recreational noise.

	Sex	Age (years)	Exposure duration	Hearing loss laterality	Hearing loss degree	SRT (dB)	IPRF (%)	Recovery	Drug use
Case 1	M	20	4 h	Bilateral	Moderate	55 and 55	80 and 80	No	No
Case 2	M	17	12 h	Bilateral	Severe and Profound	75 and 80	12 and 40	No	Yes
Case 3	M	17	3 h	Bilateral	Moderately severe	60	12 and 25	Partial	Yes
Case 4	F	28	4 h	Unilateral	Moderately severe	65	85	Total	No
Case 5	M	41	‒	Unilateral	Severe	75	20	No	Yes
Case 6	F	28	5 h	Unilateral	Moderately severe	60	76	Partial	No
Case 7	M	20	‒	Bilateral	Moderate	40 and 50	70 and 80	No	Yes
Case 8	M	20	‒	Unilateral	Moderate	40	‒	No	Yes
Case 9	F	19	1 h	Bilateral	Moderate and severe	65 and 80	20 and 16	Partial	‒
Case 10	M	17	‒	Bilateral	Severe	65	95	Partial	No
Case 11	F	40	3 h	Unilateral	Moderate	50	64	No	No
Case 12	M	23	‒	Bilateral	Moderate	55 and 45	40 and 56	Partial	Yes

M, Male; F, Female.

When asked about drug use, some patients preferred not to say, but half of them said they had used drugs, including alcohol, cigarettes, marijuana, ecstasy and “lança-perfume”. The type of exposure was also questioned, with a wide variety of responses, including New Year's Eve parties, funk and electronic music festivals, indoor parties and even football matches. Other parameters assessed were the time of exposure to sound and the distance from the sound source. Most patients were unable to provide this information precisely, but those who were able to respond reported an exposure time of 3–5 h, at a short distance from the sound source.

## Discussion

SSNHL induced by recreational noise has been gaining attention in the literature, especially considering the current leisure practices of adolescents and young adults. Although occupational noise exposure has long been recognized as an important risk factor for hearing health, more recently researchers have suggested that recreational noise exposures have the potential to increase the risk of hearing loss in individuals outside of the workplace.^15^, ^16^

In the present study, 70% of the individuals were male. The literature is still scarce regarding the epidemiological profile of patients who develop hearing loss secondary to recreational noise, however some authors believe that male patients are, in fact, more likely to develop this condition, considering their lifestyle habits and hobbies. Pienkowski et al. report in their study that young people are normally exposed to different sources of noise during their free time, predominantly music in nightclubs and live concerts, especially boys.^17^, ^18^, ^19^, ^20^

Exposure to recreational noise has been associated with various environments, such as nightclubs, bars, concerts, gyms, sporting events, video game rooms, and the use of personal music players, such as headphones.^21^, ^22^ In the present study, the main environments described were also concerts and parties, with one case involving a game in a soccer stadium. According to Jaroszewski & Jaroszewska, measurements taken in nightclubs in Warsaw, Poland, indicate that certain components of the musical spectrum can contribute substantially to the harmful effects of noise exposure.^23^, ^24^ A systematic review and meta-analysis by Dillard et al. assessed the prevalence of unsafe listening practices associated with Personal Listening Devices (PLDs) and exposure to noisy entertainment venues, including 33 studies with 35 datasets and 19,046 participants, estimating a pooled prevalence of 23.81% for PLD use and 48.20% for loud recreational environments, suggesting that between 0.67 and 1.35 billion young individuals worldwide exhibit signs of recreational noise-induced hearing loss.^25^

According to Balk et al., recreational exposure to noise has become increasingly significant, especially affecting adolescents and young adults.^26^, ^27^ Armitage et al. report that, currently, 1.1 billion young people between 12 and 35 years of age are exposed to excessive recreational noise, which corroborates the findings of our study, in which the average age was 24.16 years.^28^

In a case report described by Kaul V et al., we see the story of a patient who presented with sudden deafness after a spinning class. The data suggest that the risk of noise-induced hearing damage in these classes may mimic that of a nightclub.^29^ In addition to the high-intensity sound, the risk of hearing loss in these contexts may be exacerbated by the simultaneous high-intensity aerobic activity.^30^, ^31^

Regarding the permanence of hearing loss, the literature remains controversial. Neitzel R and Fligor B suggest that permanent hearing loss is possible with sufficient exposure to high noise levels.^32^ A longitudinal study on young adults exposed to recreational noise over three years found deterioration in hearing, increased temporary tinnitus, and greater noise exposure from nightclubs and music venues.^33^ The present study shows 01 case with full recovery of hearing and 03 cases without any degree of recovery, with only 01 of them being referred for cochlear implant surgery.

When asked about drug use, some patients preferred not to comment, but half of them reported having used drugs in the context of noise exposure. Several studies have reported an association between hearing loss and smoking, as well as with the use of cocaine and other drugs. Smoking can impact the auditory system through the direct ototoxic effects of nicotine or other ototoxic substances found in cigarette smoke, but the evidence to conclude this association is not yet entirely consistent.^34^ Regarding cocaine use, Qureshi et al. believe that the loss is attributed to vasoconstriction, hemorrhage, malnutrition, or a combination of these conditions.^35^, ^39^, ^40^ Other studies have described an association between hearing loss and the use of other drugs, such as heroin and opioids, although less frequent and still without strong evidence in the literature.^36^, ^37^, ^38^, ^39^, ^40^, ^41^

Exposure to recreational noise, although widely recognized as an important risk factor for hearing health, has not been given the adequate thought by the predominantly affected population as it continues to occur. Therefore, we need to enhance public policies to publicize this type of injury that leaves permanent sequelae. However, this study has limitations due to the restricted number of cases, requiring more robust studies with a larger cohort to determine more accurately the clinical behavior and the evolution of the conditions.

## Conclusion

This study demonstrates that there is an association between exposure to recreational noise and the development of sudden sensorineural hearing loss, and that the epidemiological profile of patients affected by this condition is mainly formed by adolescents and young male adults, with unilateral and binaural involvements, severe degree of hearing loss at onset, commonly associated with the use of recreational drugs, and leaving permanent hearing damage.

## CRediT authorship contribution statement

All authors contributed equally to this work. L.F.B.S., B.L.A., J.V.L.M., L.M.M and N.O.P. reviewed data from all sites and provided interpretive analysis; L.F.B.S. and B.L.A wrote the main paper. All authors discussed the results and implications and N.O.P., M.L.F.L.M., I.A.S.T. and M.C.P.S. commented on the manuscript at all stages.

## Funding

This work was supported by Fundação de Amparo à Pesquisa do Estado de São Paulo (Fapesp) and by National Council for Scientific and Technological Development (CNPq).

## Declaration of competing interest

The authors declare no conflicts of interest.
